# The Low Chamber Pancreatic Cancer Cells Had Stem-Like Characteristics in Modified Transwell System: Is It a Novel Method to Identify and Enrich Cancer Stem-Like Cells?

**DOI:** 10.1155/2014/760303

**Published:** 2014-02-10

**Authors:** Dongqing Wang, Haitao Zhu, Yanfang Liu, Qing Liu, Xiaodong Xie, Yuepeng Zhou, Lirong Zhang, Yan Zhu, Zhijian Zhang, Zhaoliang Su

**Affiliations:** ^1^The Affiliated Hospital of Jiangsu University, Zhenjiang 212001, China; ^2^Department of Histology and Embryology, Center of Clinical Medicine and Laboratory, Jiangsu University, 301 Xuefu Road, Zhenjiang, Jiangsu 212013, China; ^3^Department of Immunology, Center of Clinical Medicine and Laboratory, Jiangsu University, 301 Xuefu Road, Zhenjiang, Jiangsu 212013, China

## Abstract

Cancer stem cells (CSCs) or cancer-initiating cells (CICs) play an important role in tumor initiation, progression, metastasis, chemoresistance, and recurrence. It is important to construct an effective method to identify and isolate CSCs for biotherapy of cancer. During the past years, many researchers had paid more attention to it; however, this method was still on seeking. Therefore, compared to the former methods that were used to isolate the cancer stem cell, in the present study, we tried to use modified transwell system to isolate and enrich CSCs from human pancreatic cancer cell lines (Panc-1). Our results clearly showed that the lower chamber cells in modified transwell system were easily forming spheres; furthermore, these spheres expressed high levels of stem cell markers (CD133/CD44/CD24/Oct-4/ESA) and exhibited chemoresistance, underwent epithelial-to-mesenchymal transition (EMT), and possessed the properties of self-renewal *in vitro* and tumorigenicity *in vivo*. Therefore, we speculated that modified transwell assay system, as a rapid and effective method, can be used to isolate and enrich CSCs.

## 1. Introduction

A distinct population of cancer cells, namely, cancer stem cells had been definite and characterized in several human cancers, including pancreatic cancer [1–4]. CSCs are defined as cells that share many properties with stem cells, including self-renewal, differentiation potentials, and long lifetime. It is hypothesized that cancer stem cells should be responsible for tumors initiation, rapid growth, and resistance to therapy, recurrence, and metastasis [5, 6]. According to this hypothesis, CSCs could be a new target for monitoring, exterminating, or preventing of cancer.

To further investigate the characteristics of the CSCs, it is important to successfully isolate them from the heterogeneous tumor cells. In the past year, there have been several reports about isolating CSCs from cancer cells by using the following different techniques. First, with the characteristic of expression of specific cell surface proteins, such as cluster of differentiation 133 (CD133) and cluster of differentiation (CD44), CSCs can be isolated by flow cytometry [7, 8]. Second, recent studies confirmed that the sphere culture system is efficient in separating CSCs from many solid tumors or cancer cells lines. These studies have suggested that CSCs can be enriched in spheres when these cells are cultured in serum-free medium supplemented with the basic fibroblast growth factor (bFGF), epidermal growth factor (EGF), B27, and insulin [9–11]. Third, with the characteristic of expressing ATP-binding cassette transporters, these cells are able to pump the fluorescent dye Hoechst-33342 out of the cells, namely identify unlabeled “side population” (SP) which, is highly enriched in stem cells [12–14]. However, all the above methods had different deficiencies. Therefore, to explore a simple and effective method to enrich CSCs has become an urgent problem.

Transwell assay is the most frequently used in method *in vitro *to access migration ability of cells [15, 16]. This assay system included two compartments: the upper and lower chamber. Cells can migrate from the upper chamber to lower chamber through the porous membrane under some chemokine existence in the lower chamber. Furthermore, the CSCs had the potential migration capabilities. In view of this, in the present study, we modified transwell assay system and recultured the lower chamber cells and studied their biology. Meanwhile, we established an approach for isolation and enrichment of CSCs *in vitro* from human Panc-1 cell lines.

## 2. Materials and Methods

### 2.1. Cell Line and Cell Culture

The pancreatic cancer cell line (Panc-1, purchased from Cell Bank of China Academy of Sciences, Shanghai, China) was cultured in DMEM-F12 (Gibco, USA) supplemented with 10% fetal bovine serum (FBS, Gibco, USA), 100 U/mL penicillin, and 100 U/mL streptomycin, in a humidified atmosphere of 95% air with 5% CO_2_ at 37°C. Cells were passaged with 0.25% trypsin/EDTA every 3 days. The bulk Panc-1 cells were chosen as the control group.

### 2.2. Modified Transwell Assay

We mixed the same volume of the DMEM-F12 supplemented with 10% FBS and agarose solution which was used to simulate the basement membrane (Invitrogen, NY, USA). Before the experiment, the upper part of the transwell chamber was precoated with mixture (0.5 mL per hole) until the liquid solidified at the normal temperature. A total of 5 × 10^5^ cells (in 200 *μ*L DMEM-F12 supplemented with 10% FBS) were seeded into the upper part of the transwell chamber (transwell filter inserts in 6.5 mm diameter with a pore size of 5 Am; Corning Incorporated, Corning, NY, USA). In the lower part of the chamber, 600 *μ*L DMEM-F12 supplemented with 10% FBS was added. Then, the transwell chamber was put in a humidified atmosphere of 95% air with 5% CO_2_ at 37°C. After 48 h, cells from lower chamber were harvested by trypsinization. The spherical clusters of cells grown under these conditions were named the lower chamber cells. And the rest of the cells left in the upper chamber were named the upper chamber cells.

### 2.3. Sphere Formation Assay

For the sphere formation assay, the bulk Panc-1 cells, the upper chamber cells, and the lower chamber cells were maintained in DMEM-F12 with 10% FBS supplemented with 20 ng/mL epidermal growth factor (Peprotech, Rocky Hill, NJ, USA), 20 ng/mL basic fibroblast growth factor (Peprotech, Rocky Hill, NJ, USA), B27 (Invitrogen Life Technologies, Carlsbad, CA, USA), and 5 ng/mL insulin seeded into 24-well plates (Corning Incorporated, Corning, NY, USA) at a low density of 20 cells/L and the number of generated spheres was counted after 5 days of culture.

### 2.4. Chemoresistance Assay

For the chemosensitivity assay, the bulk Panc-1 cells, the upper chamber cells, and the lower chamber cells were treated with or without gemcitabine that was used widely in the clinic [17]. The drug was added to the cultures for 5 days at the concentrations of 1000 ng/mL. The liquid of the gemcitabine was prepared as described previously [10]. Cell viability was evaluated by MTT assay after 5 days of drug treatment.

### 2.5. Western Blot Analysis

The bulk Panc-1 cells, the upper chamber cells, and the lower chamber cells lysates were subjected to sodium dodecyl sulfate-polyacrylamide gel electrophoresis and transferred to polyvinylidene fluoride membranes (Merck Millipore, USA). Membranes were blocked with 5% (w/v) bovine serum albumin (BSA) in TBST for 1 h at room temperature and incubated overnight with primary antibodies at 4°C. They were subsequently incubated with horseradish peroxidase-conjugated second antibodies. The immunoreactive bands were detected by chemiluminescence (ECL Plus, Merck Millipore) and relevant blots were quantified by densitometry using LANE-1D software. For immune detection, the primary antibody preparations used were as follows: rabbit-anti-human-CD24, rabbit-anti-human-ESA, and rabbit-anti-human- Bmi-1 were obtained from Santa Cruz Biotechnology (SantaCruz, CA, USA). Rabbit-anti-human-Oct-4, rabbit-anti-human-E-ca, rabbit-anti-human-Vimentin, rabbit-anti-human-N-ca, rabbit-anti-human-SHH, and rabbit-anti-human-*β*-catenin were obtained from Cell Signaling Technology (Boston, USA). Anti-*β*-actin was obtained from Abcam Company (Cambridge, Britain). The secondary antibody preparations either anti-rabbit or anti-mouse were purchased from Boster Biotechnology Company (Wuhan, China).

### 2.6. Real-Time PCR

Real-time quantitative PCR was carried out with SYBR Green qPCR SuperMix (Bio-Rad) using the CFX-96 system (Bio-Rad). Total cellular RNA was isolated using TRIzol reagent, and cDNA was synthesized from 1 *μ*g of total RNA using oligo-dT and Moloney murine leukemia virus reverse transcriptase (Toyobo, Japan). Relative expression levels of the genes were calculated using the 2^−ΔΔCT^ method.

### 2.7. Flow Cytometry Analysis

To quantify pancreatic cancer stem-like cells in the bulk Panc-1 cells and lower chamber cells, we measured the expression of the stem cells related molecular marker CD133/CD44 using anti-CD133-PE (Miltenyi Biotech Ltd., Surrey, UK) and anti-CD44-PE (BD Pharmingen, USA). Cells were harvested, disaggregated to a single cell suspension, and stained as described previously [18].

### 2.8. Analysis of Tumorigenicity *In Vivo*

Animal studies were approved by the Committee on the Use of Live Animals for Teaching and Research of the Jiangsu University. BALB/c nude mice (purchased from The Compare Medicine Center, Yangzhou University, China) were maintained under standard conditions according to institutional guidelines. Nine mice divided into three groups were employed in this study. The cell suspension of the bulk pancreatic cancer cells and the lower chamber cells was prepared; 100 *μ*L of the liquid was injected subcutaneously in each mouse with different cell numbers from 1 × 10^4^ and 1 × 10^5^ to 1 × 10^6^ cells to comparatively evaluate their tumorigenic potential. The whole study lasts for 6 weeks. The size and counter of the tumor were observed. The tumor size was determined with the product of length of the longest and the shortest diameter of the tumor by means of vernier caliper. All the data were obtained from three independent experiments.

### 2.9. Statistical Analysis

The significances of differences between groups were analyzed using one-way or multiway classification ANOVA. Values of *P* < 0.05 were considered to be significant. All experiments were performed at least in triplicate.

## 3. Results

### 3.1. The Lower Chamber Cells More Easily Form Sphere

**Figure 1:**
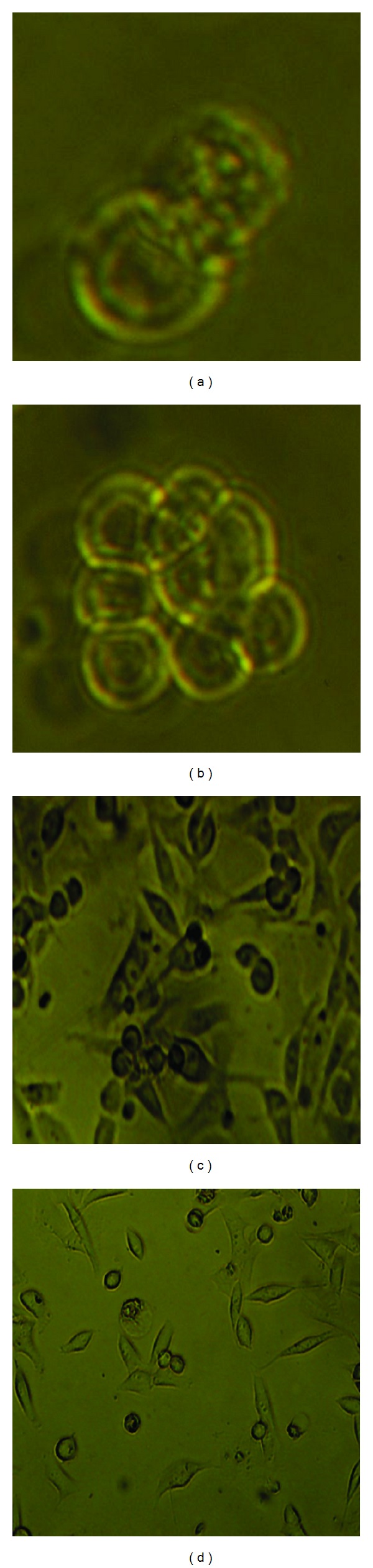
The sphere formation from the lower chamber cells. ((a), (b)) Phase-contrast images of the spheres from the lower chamber cells cultured in DMEM-F12 containing 10% FBS from day 1 to day 5 ((a) for day 2, (b) for 5). The size of the sphere increased in a time-dependent manner. ((c), (d)) Phase-contrast images of the cells from the bulk pancreatic cancer cells and the upper chamber cells cultured in DMEM-F12 containing 10% FBS in the fifth day. No spheres can be detected. (c) was the bulk pancreatic cancer cells, while (d) was the upper chamber cells group. Scale bar equal to 50 *μ*m.

When the total of 5 × 10^5^ pancreatic cells was seeded into the upper part of a transwell chamber, about 1 × 10^3^cells can pass in lower chamber after 48 h and reach the maximal levels which was confirmed by the Giemsa assay (data not shown). The bulk Panc-1 cells, the upper chamber cells, and the lower chamber cells were dissociated into single cells and seeded into the same culture medium. The lower chamber cells aggregated and differentiated into three-dimensional (3D) balls with a spheroid configuration DMEM-F12 containing 10% FBS. The size of the sphere increased in a time-dependent manner by dynamic observation (Figures 1(a) and 1(b)). Then, the spheres were dissociated into single cells and passaged in the same medium, but the tumor spheres reformed 5 days later. The spheres with a tight, round, and smooth contour were observed. However, the bulk Panc-1 cells and the upper chamber cells grew as adherent cells in DMEM-F12 containing 10% FBS, and even after several passages, there were no spheres detected (Figures 1(c) and 1(d)).

### 3.2. The Percentage of Cd133^+^ Cd44^+^ Subpopulation Was Higher in the Low Chamber Cells

**Figure 2:**
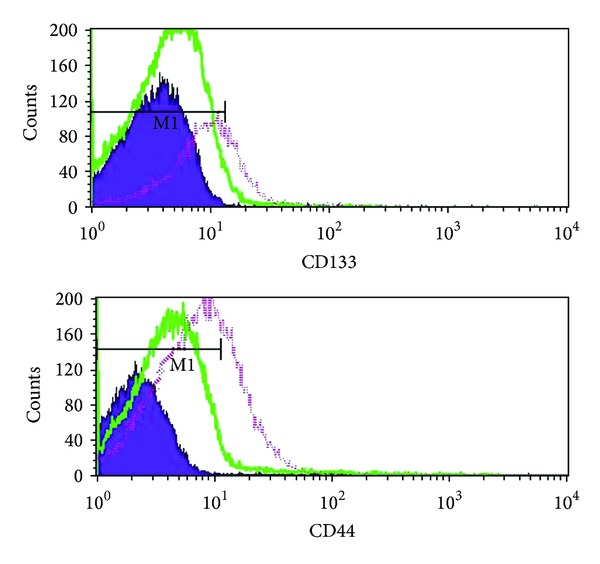
The CD133+ and CD44+ subpopulations were enriched from the lower chamber cells. The percentage of CD133+ subpopulation (a) and CD44+ subpopulation (b) was analyzed by flow cytometry. The pink color line represents the lower chamber cells group, while the green line represents the bulk pancreatic cells, and the blue area represents the isotype control.

It was widely accepted that the surface markers CD133 and CD44 have been well defined for isolating CSCs from pancreatic adenocarcinomas. Due to their increased tumorigenicity, clonogenicity, and metastatic potential, the CD133^+^ and CD44^+^ subpopulation isolated from Panc-1 cells were considered to own the properties of stem cells. By flow cytometry analysis, we sought to quantify the CD133^+^ and CD44^+^ subpopulation in the lower chamber cells and the Panc-1 cells, respectively. The results demonstrated that the proportion of CD133^+^ was much lower in the bulk Panc-1 cells population (3.23 ± 0.47%) than in the lower chamber population (38.6 ± 3.10%; 18-fold higher percentage) (Figure 2(a)). And the percentage of CD44^+^ cells population was 9-fold higher in the lower chamber cells (34.88 ± 2.12%) than in the bulk Panc-1 cells population (4.73 ± 0.47%) (Figure 2(b)). Collectively, the stem-like pancreatic cancer cells were enriched in the lower chamber cells.

### 3.3. The Low Chamber Cells Population Highly Expressed Cancer Stem-Like Cell Markers

**Figure 3:**
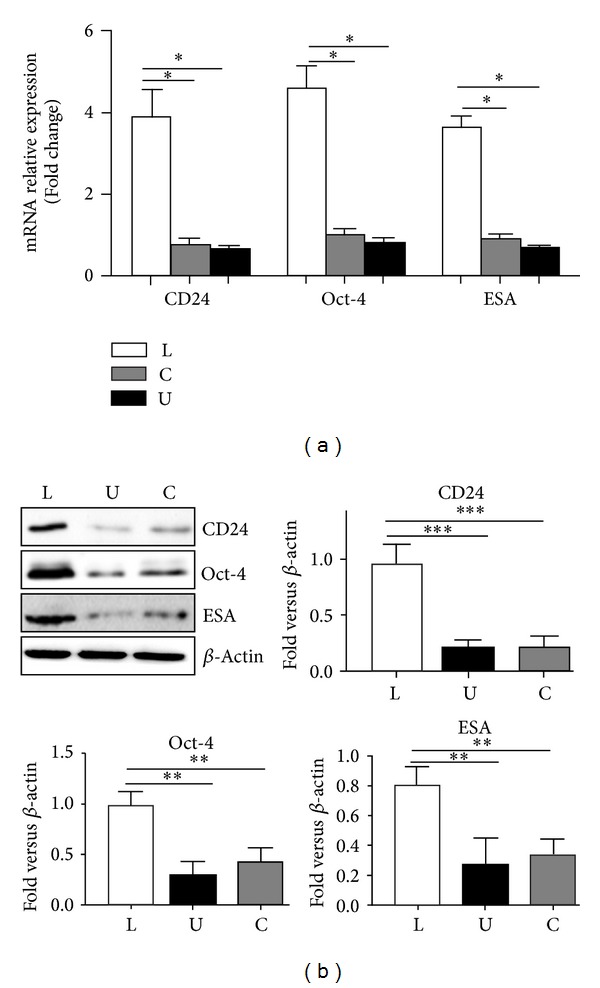
Comparison of the expression level of stem cell related genes and proteins in the three groups cells. (a) CD24, Oct4, and ESA genes expression in the lower chamber cells, bulk Panc-1 cells, and the upper chamber cells were detected by RT-PCR. (b) Expressions of CD24, Oct4, and ESA proteins in the lower chamber cells and Panc-1 cells were detected by Western blot. Data were normalized to *β*-actin levels. Experiments were repeated three times with similar data. L represents the lower chamber cells; C represents the bulk pancreatic cells; U represents the upper chamber cells (*P < 0.05, **P < 0.01, and ***P < 0.001).

Oct-4 (octamer-binding transcription factor 4) is a critical transcription factor for maintaining the survival of cancer stem like cells as well as the pluripotent state of stem cells, through a highly complicated signaling network. CD24 (cluster of differentiation 24) and ESA (epithelium specific antigen) were also used as special markers of cancer stem cells. Furthermore, the expression level of Oct-4, CD24, and ESA was analyzed in lower chamber cells. The mRNA levels of CD24, Oct-4, and ESA were significantly increased in lower chamber cells compared with parallel bulk Panc-1 cells and the upper chamber cells (Figure 3(a)). And the same data was also further confirmed by protein levels (Figure 3(b)).

### 3.4. The Lower Chamber Cells Had the Epithelial-to-Mesenchymal Transition (EMT) Potentiality

**Figure 4:**
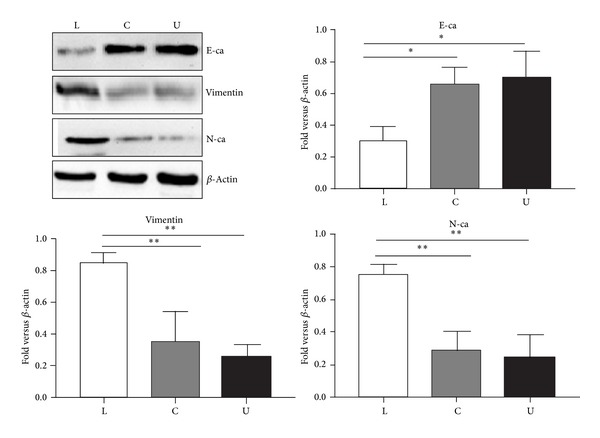
The lower chamber cells demonstrate undergoing EMT. The expression levels of E-cadherin, Vimentin, and N-cadherin in the lower chamber cells, the bulk Panc-1 cells, and the upper chamber cells were determined by Western-blot analysis. Densitometry analysis revealed the differences between the lower chamber cells, bulk Panc-1 cells, and the upper chamber cells. Data were normalized to *β*-actin levels. Experiments were repeated three times with similar data. L represents the lower chamber cells; C represents the bulk pancreatic cells; U represents the upper chamber cells (*P < 0.05, **P < 0.01, and ***P < 0.001).

Epithelial-to-mesenchymal transition (EMT) is a cellular process during which epithelial cells lose their polarized organization and cell-cell junctions, undergo changes in cell shape and in cytoskeletal organization, and acquire mesenchyme characteristics and increased cell migration and invasion. EMT involves the loss of epithelial markers, such as the adherent's junction proteins E-cadherin. Concomitantly, a number of mesenchymal markers are increased in their expression, including N-cadherin, Vimentin. The concepts of CSCs and EMT address key aspects of tumorigenesis, growth, and metastasis. Recently, EMT was shown to be associated with the CSCs phenotype in various solid tumors. We further investigated whether the stem-like lower chamber cells exhibit EMT. Western blot analysis showed that the protein level of the epithelial marker E-cadherin was decreased. However, Vimentin and N-cadherin increased in the lower chamber cells compared with the bulk Panc-1 cells and the upper chamber cells which indicated that the lower chamber cells had undergone EMT (Figure 4).

### 3.5. The Lower Chamber Cells Were Resistant to Gemcitabine Than Panc-1 Cells

**Figure 5:**
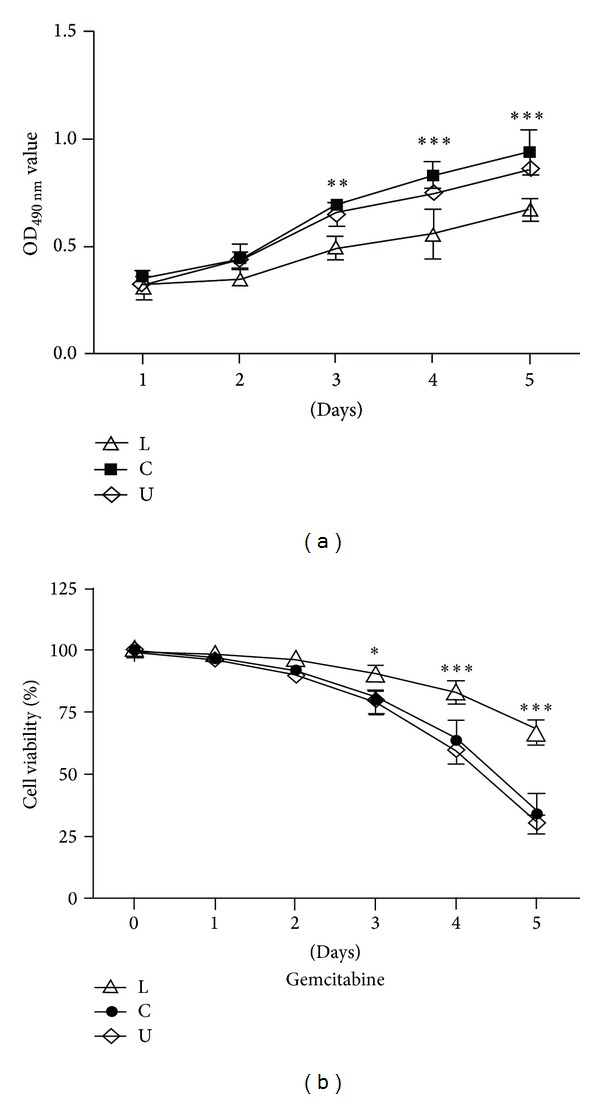
High resistance to gemcitabine in lower chamber cells. Under the condition without gemcitabine, the cell viability of the bulk pancreatic cells was higher than those of the lower chamber cells and the upper chamber cells. The proliferation ability of the three groups' cells was inhibited by gemcitabine. However, the survival rate of lower chamber cells was significantly higher than those of the bulk pancreatic cells and the upper chamber cells. Significant differences in chemosensitivity were observed among the lower chamber cells, the bulk pancreatic cells, and the upper chamber cells (*P < 0.05, **P < 0.01, and ***P < 0.001).

Resistance to chemotherapy is another property that can distinguish CSCs from other cancer cells. In order to identify the drug sensitivity differences among the lower chamber cells, the upper chamber cells, and the bulk Panc-1 cells, gemcitabine was used to treat the three types of cells. The cells viability of the bulk Panc-1 cells was much higher than the lower chamber cells and the upper chamber cells without gemcitabine. However, MTT assays demonstrated that the proliferation of the lower chamber cells was inhibited by gemcitabine and the survival rate of lower chamber cells was significantly higher than the bulk Panc-1 cells and the upper chamber cells (*P* < 0.05) (Figure 5(b)). The results suggested that the lower chamber cells showed more gemcitabine resistance ability than the Panc-1 cells and the upper chamber cells.

### 3.6. Self-Renewal Pathways Related Proteins Are Upregulated in Lower Chamber Cells

**Figure 6:**
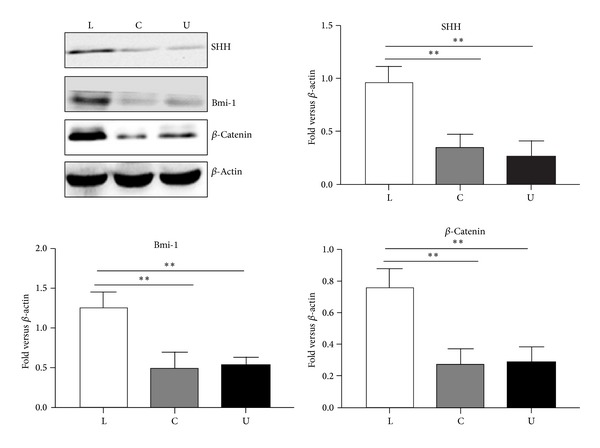
The lower chamber cells displayed high level of self-renewal related proteins. The expression levels of SHH, Bmi-1, and *β*-catenin in the lower chamber cells, the bulk Panc-1 cells, and the upper chamber cells were determined by Western-blot analysis. Densitometry analysis revealed the differences between the lower chamber cells, bulk Panc-1 cells, and the upper chamber cells. Data were normalized to *β*-actin levels. Experiments were repeated three times with similar data. L represents the lower chamber cells, C represents the bulk pancreatic cells, U represents the upper chamber cells (*P < 0.05, **P < 0.01, and ***P < 0.001).

Several developmental signaling molecules have been implicated in the self-renewal process of normal stem cells, including Notch, hedgehog, and Wnt. Deregulation of these signaling molecules has been associated with stemness of cancer cells and tumorigenesis. We next determined if there was increased expression of the developmental signaling molecule SHH, Bmi-1, or *β*-catenin in the lower chamber cells population. Western blot showed that the SHH, Bmi-1, and *β*-catenin were overexpressed in the lower chamber cells compared to the Panc-1 cells and the upper chamber cells (Figure 6).

### 3.7. The Lower Chamber Cells Had High Tumorigenic Characteristics

**Figure 7:**
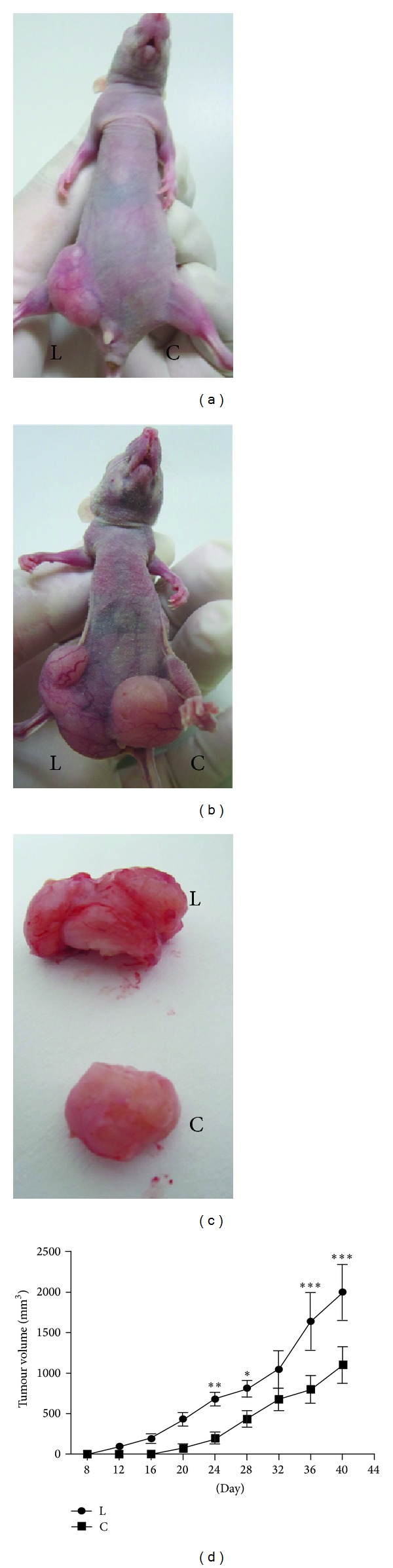
Comparison of newly generated tumors between the lower chamber cells and parental Panc-1 cells in BALB/c nude mice. (a) 1×10^4^ of the lower chamber cells and the bulk Panc-1 cells were injected into BALB/c nude mice on the right and left flanks, respectively. The lower chamber cells formed tumors 2 weeks later, while Panc-1 cells failed. (b) 1 × 10^6^ of the lower chamber cells and the bulk Panc-1 cells were injected into BALB/c nude mice on the right and left flanks, respectively. Both of the two groups formed tumors 2 weeks later. (c) Gross appearance of a representative tumor formed by the same number (1 × 10^6^) of bulk Panc-1 cells and the lower chamber cells into BALB/c nude mice. (d) Growth curves showed that the growth speed of the lower chamber cells group was much faster than their parallel Panc-1 cells. L represents the lower chamber cells; C represents the bulk pancreatic cells (*P < 0.05, **P < 0.01, and ***P < 0.001).

Uptill now, the tumorigenic capacity *in vivo* is still considered to be a method for measuring CSCs. To address the lower chamber cells tumorigenic characteristics, the same number of the bulk Panc-1 cells and lower chamber cells was injected into BALB/c nude mice, respectively. It was easy to be found that tumors can be detected in the lower chamber cells group when the cells number reached 1 × 10^4^ in two weeks. However, no tumors formed in the Panc-1 cells group until the cells number up to 1 × 10^6^ (Figures 7(a) and 7(b)). Furthermore, when the same number of cells (1 × 10^6^) was injected into BALB/c nude mice, the contour of the tumors yielded from the lower chamber cells was irregular and grown in the invasion way; additionally, the volume of the tumor originated from lower chamber cells was larger than their parallel Panc-1 cells (Figure 7(c)). Also, the growth speed of the lower chamber cells group was much faster than its parallel Panc-1 cells (Figure 7(d)). All the results indicated that the lower chamber cells had high tumorigenic characteristics and might be enriched in cancer stem cells.

## 4. Discussion

Over the past decades, intensive research brought forth a deeper understanding of pancreatic carcinoma formation and outgrowth, giving rise to sophisticated therapies of suffering patients. However, the overall prognosis of pancreatic cancer is still very poor, with the 1-year survival rate of less than 20% and a 5-year survival rate of less than 5% [19–21]. Accumulating data support the concept that the capability of the tumor to grow and propagate is dependent on a small subset of cells within a tumor, termed cancer stem cells and/or progenitor cells. CSCs represent a small population of cancer cells that exhibit self-renewal and differentiation characteristics. Also, CSCs are responsible for tumor formation, progression, drug resistance, metastasis, and recurrence. So, it is important to identify and isolate cancer stem cells. At present, CSCs have been isolated from various solid tumors including pancreatic cancer [2, 3, 22–24].

Currently, identification of pancreatic CSCs has been achieved successfully via using the following different techniques: (i) by flow cytometry analysis, cancer stem cells can be identified mainly based on the special surface markers. CD34 and CD38 were used as markers in the original studies of leukemia stem cells which were first identified at the beginning of research [25]. Subsequently, CD133, CD44, CD24 were selected as CSC markers in many solid tumors, including pancreatic carcinoma. However, this method had some obvious defects [26, 27]. Firstly, there is no apparent consensus regarding the standard marker to be used for the identification of CSCs in pancreatic cancer. Secondly, the technology is not amenable to assess more than two markers on a single cell. Thirdly, the proportion of CD133^+^CD44^+^ cells acquired in this way is only approximately 0.1–10% of the total population. So, insufficient cell numbers severely limit research on CSCs. (ii) By exploiting functional characteristics, sorting the side populations of cancer cells via intracellular Hoechst-33342 exclusion cells has also been used for the identification and enrichment of CSCs, called SP technique. The cells that exclude Hoechst dye are SP cells. Because the SP cells express high levels of stem cell markers and low levels of differentiating markers, the SP subpopulation is widely used to enrich stem cells [12, 28]. It is the most widely used strategy to identify stem-like cells in cancer cell culture in one time. The impracticality of all the above methods for CSCs was as follows: firstly, SP method was inefficient (0.23%–12%) for further research. Meanwhile, the SP or non-SP cells showed similar characteristic in clonogenic and tumorigenic capacity in some cell lines. Secondly, Hoechst-33342 staining can affect cell differentiation [29–31]. Thirdly, because of the characteristic of antianoikis and growth in the anchor independent manner, some researchers have also suggested that CSCs can be enriched when the cancer cells cultured in serum-free medium are supplemented with special growth factors. This method is easy and does not need special instrument, but the method also has defects [32, 33]. One was that the morphology of the spheres displayed irregular, loose and it is more likely that the spheres are aggregates of the cells rather than clone; the other was that it is a time-consuming process, because the formation of the spheres usually needs 3 weeks; the third was that the cells in center of sphere often undergo degeneration or apoptosis; additionally, the supplemented materials were expensive. Compared with the methods mentioned above, modified transwell assay isolated cancer stem cells that are only dependent on cells' migration. This method had more obvious advantages: (1) the percentage of CD133^+^CD44^+^ cells was obviously increased. Our results clearly showed that the percentage of CD133^+^CD44^+^ cells in the lower chamber cells can reach 30%; any method mentioned above was not up to the level; (2) the obvious stem cells characteristics. Our results demonstrated that the lower chamber cells were greatly different from the bulk pancreatic cancer cells and the upper chamber cells. The lower chamber cells were more easily spheric, resistant to gemcitabine, self-renewing, highly expressed CD24, Oct4, and ESA, and of high tumorigenic characteristics [34–39]. Additionally, this method did not affect the cell inherent characteristic. (3) Simple and convenient, wide application. This method did not need special instrument and was simple and convenient. Furthermore, we also used this method to isolate the CSCs of human gastric cancer cells, SGC7901, and human hepatic cancer cells, HCCLM3 (the data were not shown). The similar results were obtained. All these results show that modified transwell assay may be used widely in some cancer stem cells sorting.

In conclusion, modified transwell system, as a simple, reliable, and reproducible method, could be used to isolate some different sources of tumor stem cells.
